# Physical activity level among male and female middle to high school students and impact of perceived school climate: a longitudinal analysis

**DOI:** 10.3389/fpubh.2025.1582693

**Published:** 2025-07-18

**Authors:** Biplav Babu Tiwari, Jacob Matta, Michael R. Thomsen, Linlin Da, Kiran Thapa, Ye Shen, Justin B. Ingels, Janani Rajbhandari-Thapa

**Affiliations:** ^1^Department of Health Policy and Management, College of Public Health, The University of Georgia, Athens, GA, United States; ^2^Department of Epidemiology and Biostatistics, College of Public Health, The University of Georgia, Athens, GA, United States; ^3^Department of Health Policy and Management, Fay W. Boozman College of Public Health, University of Arkansas for Medical Sciences, Little Rock, AR, United States

**Keywords:** adolescents, adolescent health, longitudinal study, physical activity, school climate

## Abstract

**Background:**

Physical activity levels are declining among middle and high school students, and schools could provide an ideal space for engaging adolescents in physical activity. The study aimed to assess physical activity levels among male and female middle to high school students and the impact of perceived school climate.

**Methods:**

This longitudinal study utilized the Georgia Student Health Survey (GSHS) from 2016 to 2020, with change in the proportion of physically active students as an outcome and school climate measures as predictors. Factor analysis yielded a composite index for each school climate measure. Descriptive analysis measured the trend and male vs. female differences in physical activity. A multivariable linear regression model was developed to assess the impact of school climate on physical activity and male–female differences.

**Results:**

The decline in physical activity with increasing grade levels was notably more pronounced in females than males. Improvement in the perception of school climate measures was consistently linked to an increased proportion of physically active students as they progressed to higher grades (for example, change in perception of school connectedness: *β* = 5.39; *p* < 0.001).

**Conclusion:**

The study findings underscore the importance of fostering positive school climates to mitigate the decline in physical activity levels, especially among adolescent females.

## 1 Introduction

Inactivity is a global concern; 3 in 4 adolescents (11–17 years old) do not meet the World Health Organization (WHO) recommended 60 min (mins) or more of moderate-to-vigorous intensity physical activity (MVPA) (1, 2). Regular physical activity among adolescents could improve cardiorespiratory fitness, muscle mass growth, metabolic syndrome, and mental health (3–6). Regardless of the myriad benefits of being physically active, studies report a declining trend of physical activity among adolescents (7, 8). A study by the WHO among 1.6 million adolescents reported that 81% of 11-17-year-old school children are not meeting the recommended level of physical activity (7). Further, recent studies have reported that a higher proportion of female adolescents are not meeting the recommended physical activity levels compared to male adolescents (9, 10). A longitudinal study found a significant decline in mean minutes (mins) of MVPA between prepubertal and pubertal periods from 52.1 min to 42.6 min for girls, compared to 61.4 min to 49.3 min for boys (11). Such a decline in physical activity during the transition from childhood to adolescence is particularly concerning as many studies continue showing a positive impact of physical activity on physical and mental health (12–14). For example, a higher frequency of physical activity among adolescents was significantly associated with reduced odds of mood disorder, bipolar II disorder, and general psychological distress (5). There is a need to identify new areas of population health-based interventions in large settings to address global inactivity.

Schools could provide that opportunity for engaging adolescents in physical activity, as they offer structured settings and social opportunities that can significantly influence students’ behavior (15). According to Social Cognitive Theory, individuals are influenced by both personal and environmental factors, suggesting that the school climate, a key environmental factor, can shape students’ physical activity levels (16). A study by Owen et al. found that 73.7% of adolescents want to get involved in physical activity at school, and 88.8% want to do such physical activities with their friends (17). This indicates that peer relationships, a critical component of school climate, can compound students’ motivation to participate in physical activity. Further, schools can foster a motivational environment that prioritizes personal improvement and effort over competition (18). Such an environment fosters a supportive and inclusive atmosphere that could encourage all students to participate in physical activities, regardless of their skill levels (19). A study by van Sluijs et al. found that a school climate with a motivational environment and comprehensive school-based physical activity programs was associated with improved physical activity levels among students (20). These school climates include school support environment (rules and adult guidance present at school), school connectedness (likeness to school, feeling of fit and success, and connection with others at school), peer social support (connection with other students in the school), school physical environment (care and conditions for the school), cultural acceptance (perception of diversity acceptance at school), school safety (self-perceived safety of school, occurrence, and involvement in a fight at school), peer victimization (extent of bullying by peers), and adult social support (student’s perception of teachers’ attitude toward the students) (21).

A review by WHO found that school-based programs, such as increased physical education, active classrooms, and regular sports activities, significantly improved physical activity levels and academic achievement (22). This is corroborated by observational and experimental studies, which consistently demonstrate the positive impact of a supportive school climate on students’ physical activity levels and academic achievement (23–28). Further, studies have reported that school climate-related barriers, such as a lack of social support from friends and teachers, accessible places, and professional physical education teachers, are associated with declining physical activity levels among students (29, 30). The evidence strongly suggests that a positive school climate can significantly influence students’ physical activity levels. However, to our knowledge, there is no longitudinal research on the impact of school climate on adolescents’ physical activity levels. This study extends the existing body of knowledge on the association of school climate with student physical activity.

In the United States (US), the Centers for Disease Control and Prevention’s (CDC) recommendation is consistent with the WHO recommendation of 60 min of MVPA (31), however, physical activity is consistently decreasing with age among adolescents in the United States (32) as it is globally. In the United States, the state of Georgia (GA) has high differences in physical activity levels among male vs. female adolescents; a majority (57%) of high school male students were physically active compared to only 35% of their female counterparts in 2017 (21). GA is also a state in the southern US with high rates of sedentarism among adolescents; 15.4% of children aged 5–17 are obese, 24% of high school students do not meet their 60 min of MVPA requirement, and 41.2% report using a computer for three or more hours for something other than related to school work (8). Thus, we base this study in Georgia to assess physical activity levels among male and female middle to high school students and the impact of perceived school climate.

## 2 Methods

### 2.1 Participants and study design

This study followed a longitudinal design to study the impact of school climate on adolescents’ physical activity (PA) levels using secondary data from an anonymous survey of middle and high-school students—the Georgia Student Health Survey (GSHS) 2.0 from 2016 through 2020. The GSHS is a standardized annual statewide anonymous survey developed by the Georgia Department of Education (GDoE) in collaboration with the Georgia Department of Public Health (GDPH) and research universities, and has been administered since 2001 by the GDoE (33). The GSHS survey is primarily used for School Climate Star Ratings for all Georgia Public Schools; participation is mandatory for public schools with passive parental permission and is optional for private schools (34, 35). The data is collected every school year from October to February; as such, the data for a calendar year also includes the last quarter of the previous year (35).

The data setup allowed longitudinal tracking of physical activity levels and school climate separately for male and female students within each grade as they advanced in grade (from 6th to 12th grade) within each participating school. Individual student responses were aggregated up to the class level separately for male and female students using the grade variable to aggregate individual responses. GSHS is an anonymous survey, and even though longitudinal analysis at the individual level is impossible, it provides opportunities for longitudinal analysis at the grade level. The students that make up a grade cluster could change year over year, but the study assumes that the cluster behavior does not change over time. The assumption is based on the homogeneity of the student body served by school districts in the US, where home addresses govern which school students go to, and homes in a school district have similar social determinants of health (36, 37).

Under GA code 20–2-291, high schools in GA contain any grade above grade eight, and middle school contains no grade below grade four or above grade eight (38). This standard definition of middle and high school grades helped to ensure uniformity across Georgia schools for setting up the data for longitudinal analysis. Grades four and five were excluded from the study data because GSHS collects data on those grades using an elementary school survey with limited questions on school climate measures. Amidst COVID-19, a shortened version of the “Voluntary Student Wellness Survey” was conducted, which replaced the GSHS 2.0 for 2021 and 2022 (34). Therefore, this study did not include data from 2021 and 2022. Data collection for 2020 was conducted from October 2019 to February 2020, and the school closure due to COVID-19 in Georgia started on March 18, 2020 (39). Therefore, COVID-19-related school closures had no impact on the response rate in 2020. The study data included 3,423,888 middle and high school students (51.25% females) from the schools with at least two consecutive years of data across the study period (2016–2020).

### 2.2 Study measures

We used self-reported PA and school climate measures from the GSHS. Specifically, the measure of PA was based on the response to the following question: “In the past 7 days, how many days were you physically active for at least 60 min at school or home?” with possible responses “Not at all,” “1 day per week,” “2–3 days per week,” and “4–5 days per week.” Though the highest response level of “4–5 days per week” does not completely align with the aforementioned WHO and CDC’s recommendations for PA for youth in this age group (2, 31), previous studies (21, 40, 41) have used it to define being physically active. Given this limitation, we dichotomized the responses into two categories: physically active (coded 1 if responded “4–5 days per week”) and less physically active (coded 0 for all other responses), instead of using met/not met the physical activity requirement.

#### 2.2.1 Longitudinal outcome measure: change in physical activity

The outcome measure was the change in the proportion of physically active male and female students ( $\Delta N_{m/\text{fGYS}}^{p}$ ) as they advance in grade level, as illustrated in Equation 1. The change subtracts a lower grade (for example, grade 7^th^) from a higher grade (for example, grade 8^th^); therefore, a negative value in the change shows a decline in the proportion of physically active students (for example, if $N_{\text{female},7\mathrm{th}\;\text{grade},2018,\text{in school}\;S}^{p}$ is 0.6 and $N_{\text{female},8\mathrm{th}\;\text{grade},2019,\text{in school}\;S}^{p}$ is 0.5, the $\Delta N_{m/\text{fGYS}}^{p}$ by definition is −0.1).


(1) $$\Delta N_{m/\text{fGYS}}^{p}=(\frac{\sum {\left(P_{m/f}(G+1)(Y+1)\right)}_{S}}{\sum {\left(T_{m/f}(G+1)(Y+1)\right)}_{S}}-\frac{\sum {\left(P_{m/f}\mathrm{GY}\right)}_{S}}{\sum {\left(T_{m/f}\mathrm{GY}\right)}_{S}})\times 100$$

$P_{m/f}$ and $T_{m/f}$ the number of physically active and total number of male and female students, respectively, in grade G in school year Y within school S; m/f = female/male, G = Grades 6th to 12th, and Y = 2016 to 2020.

#### 2.2.2 Measures of school climate

Eight measures of school climate: school connectedness, peer social support, adult social support, cultural acceptance, physical environment, school safety, peer victimization, and school support environment were used, based on previous studies (21, 42–48). Students’ perception of each school climate measure was assessed on a four-point Likert scale, where a higher score denotes a higher level of agreement on perceptions, i.e., 1 = strongly disagree, 2 = somewhat disagree, 3 = somewhat agree, and 4 = strongly agree. The questions on school climate were measured across all study years (2016–2020). However, the questions were revised in 2019, resulting in the exclusion of 6 questions out of 44 questions that were assessed during 2016–2018. Our study is based on the 38 questions consistently asked across all study years (questions related to the study are presented in Appendix Table A1). We reordered some responses (identified in Appendix Table A1 with an asterisk) to ensure consistency across all questions such that positive responses were assigned higher values. For example, in the question “My school is well maintained,” the highest response (strongly agree) is positive and is thus assigned a higher value. However, for the question “I have felt unsafe at school or on my way to or from school,” the highest response (strongly agree) is negative and is reordered so that the strongly disagree (positive response) is assigned a higher value. GDoE conducted exploratory and confirmatory factor analyses during their initial validation to group the school climate-related questions into these measures (48, 49). Further, reliability assessment by GDoE using measurement invariance analyses has found these questions and their respective school climate measures to be consistent overall and across race/ethnicity and male/female students (48).

There were four to seven questions for each school climate measure. We conducted principal component factor analyses for each school climate measure to reduce the dimensionality of the eight school climate measures, following the technique used in earlier studies using GSHS data (21, 40, 48). From the four to seven questions within each school climate measure, the principal component analysis yielded a single latent factor for each school climate measure that met the latent factor selection criteria of the factor with an Eigenvalue greater than one for each measure. Appendix Table B1 outlines the Eigenvalues of each latent factor. We used the resulting measure of latent indices for each student on each school climate measure to aggregate it up to the class level separately for female and male students within each grade. Similar to the change in the proportion of physically active students (longitudinal outcome measure), the change in perception of school climate between grades by gender was obtained by subtracting the average perception of school climate measures of a lower grade from a higher grade by gender. Therefore, a negative value in the change shows a decline in school climate perception.

### 2.3 Data analysis

A descriptive analysis of the proportion of physically active male and female students by grade was conducted, combined for all years and separately for each year. Then, we tested the grade level association and differences between male and female students using a multiple linear regression model (Equation 2) with the proportion of physically active students ( $N_{m/\text{fGYS}}^{p})$ as a function of grade (G), male/female (m/f), and a categorical-by-categorical interaction term for grade and female gender. A negative coefficient on the interaction term would show a decline in the proportion of physically active students. Equation 2 was run separately for each year.


(2) $$N_{m/\text{fGYS}}^{p}=\beta_{0}+\beta_{1}m/f_{S}+\beta_{2}G_{S}+\beta_{3}{\left(G\times \text{female}\right)}_{S}$$

Second, we developed multiple unadjusted linear regression models (Equation 3) to test the association between the change in the perception of school climate measures ( $\mathrm{\Delta S}C_{m/\text{fGYS}}$ ) and change in the proportion of physically active students ( $\Delta N_{m/\text{fGYS}}^{p}$ ) for the full data and data subsets for males and females. All these models were run separately for each school climate measure.


(3) $$\Delta N_{g/\text{fGYS}}^{p}=\beta_{0}+\beta_{1}\mathrm{\Delta S}C_{m/\text{fGYS}}+\beta_{2}\mathrm{\Delta Y}$$

In these models, the Δ Y is the change in year due to grade transition (for example Grade 6 in 2016 transition to Grade 7 in 2017), i.e., 2016–2017, 2017–2018, 2018–2019, and 2019–2020.

Lastly, following a change score method (50), we developed our final multiple linear regression models (Equation 4) with the change in the proportion of physically active students as a function of the change in perception of school climate measures ( $\mathrm{\Delta S}C_{\text{gGYS}}$ ), proportion of physically active students in the previous grade (i.e., baseline), grade, male/female, and a categorical-by-continuous interaction term for male/female and change in perception of school climate measures. The models were run separately for each school climate measure.


(4) $$\begin{array}{l} \Delta N_{m/\text{fGYS}}^{p}=\beta_{0}+\beta_{1}N_{m/\text{fGYS}}^{p}+\beta_{2}\mathrm{\Delta S}C_{m/\text{fGYS}}+\beta_{3}m/f_{S}+\\ \beta_{4}{\mathrm{\Delta G}}_{S}+\beta_{5}\mathrm{\Delta S}C_{m/\text{fGYS}}\times \text{female}+\beta_{6}\mathrm{\Delta Y} \end{array}$$

In these models, ΔG is the grade transition, i.e., Grade 6-to-Grade 7, Grade 7-to-Grade 8, Grade 8-to-Grade 9, Grade 9-to-Grade 10, Grade 10-to-Grade 11, and Grade 11-to-Grade 12.

In all models with the school year, we used the school year as a fixed effect to account for possible omitted variable bias due to year-to-year changes in levels of physical activity. We also clustered the standard errors at the school level to account for possible bias as a result of school system-level factors that may affect physical activity. R version 4.1.0 was used to conduct data analysis with the RStudio integrated development environment. A *p*-value of <0.05 was used as the criterion for significance reporting.

## 3 Results

**Figure 1:**
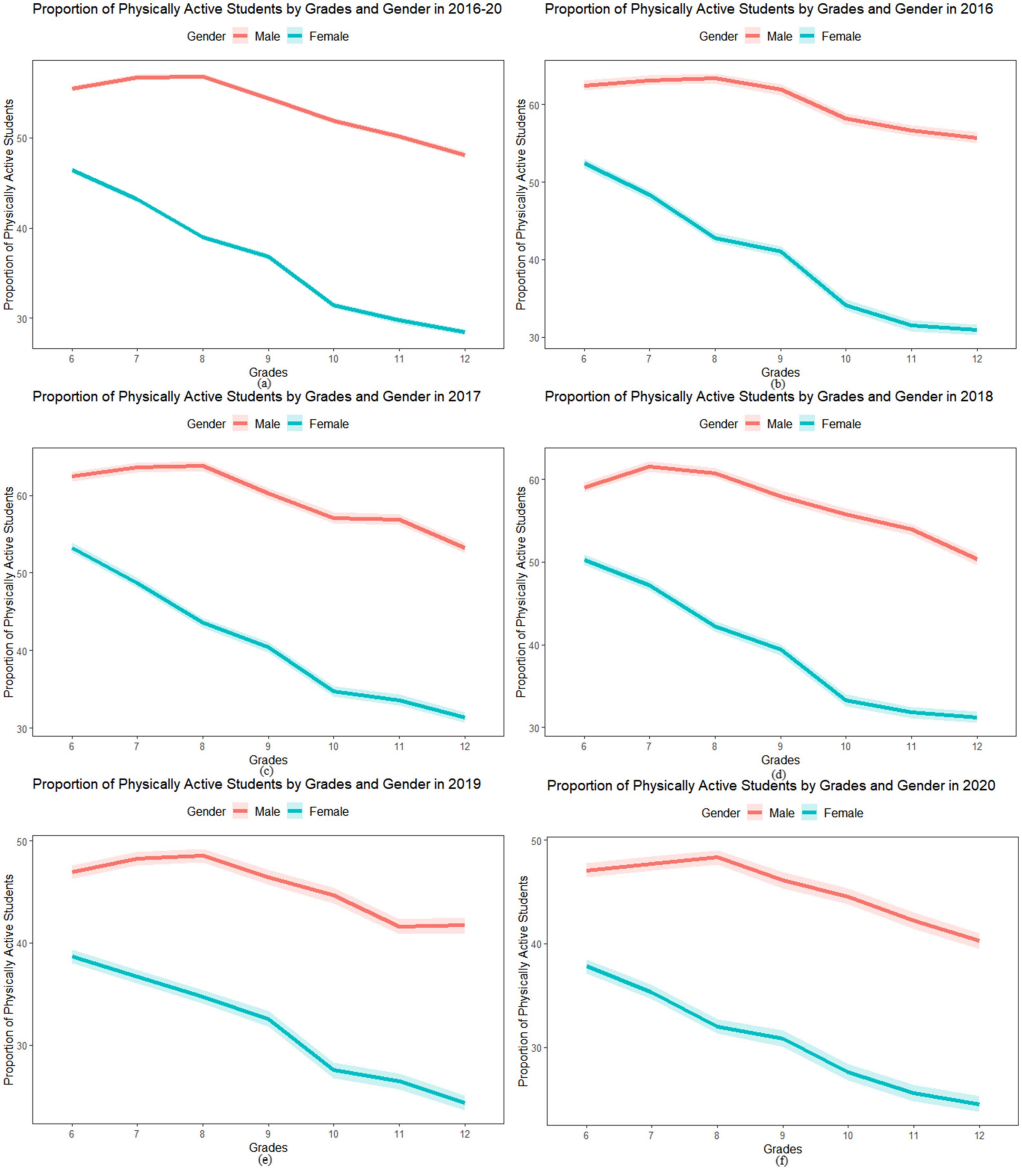
Proportion of physically active students and 95% confidence interval (CI) by gender from grade 6 to 12 for all years combined and by year **(a)** 2016 to 2020 **(b)** 2016, **(c)** 2017, **(d)** 2018, **(e)** 2019, and **(f)** 2020.

We found the proportion of students who reported being physically active decreased from grade 6 to grade 7 and so on until grade 12 across all study years. The proportion of physically active male students was higher than the proportion of physically active female students in each grade; and the decrease in the proportion of physically active students in grade 6 vs. grade 12 was higher among females compared to males, i.e., the mean (yearly) decrease from grade 6th to grade 12th for females was 17.23%, and for males was 7.87% (Appendix Table C1). Figure 1 shows that the decrease among females was significantly more pronounced compared to male students across all years combined (Figure 1a) and each year separately (Figures 1b–f). The linear regression results (shown in Appendix Table D1) confirm the widening gender gap in physical activity as adolescents advance through the grades. The results show negative and significant coefficients in grades 10, 11, and 12 compared to grade 6 across all years. Results also show a significant negative coefficient for the interaction term and generally increase from lower to higher grades for females, suggesting a higher decrease among female students compared to male students.

We found a significant positive unadjusted association between the change in the perception of school climate measures and the change in the proportion of physically active students among all students and male and female students separately, except for cultural acceptance and peer victimization (Table 1). The results suggest that with an improved perception of school climate, there is an improvement in the proportion of physically active students. The coefficients were higher for peer social support for female students (7.744, *p* value<0.001), whereas it was school connectedness for male students (8.001, *p* value <0.001).

**Table 1:** Unadjusted association of changes in the proportion of physically active students with changes in the perception of school climate measures for all students, female-only and male-only students.

Year-to-year changes in school climate measures	Year-to-year changes in proportion of physically active students		
	All students	Female-only	Male-only
Peer social support	7.755***	7.744***	7.713***
School connectedness	7.532***	7.049***	8.001***
School support environment	7.073***	7.237***	6.961***
School safety	4.944***	4.697**	5.258***
Physical environment	4.885***	5.698***	4.102***
Adult social support	3.664***	4.280***	2.992*
Cultural acceptance	2.706***	3.336**	2.036
Peer victimization	0.036	−2.062	1.764

*p < 0.05; *p < 0.01; ***p < 0.001; study years were included in the model as a covariate, and the standard errors were clustered at the school level.

The adjusted final model confirms that a change in the perception of school climate measures is significantly associated with a change in the proportion of physically active students. For example, a one-unit increase in change in the perception of school connectedness was significantly (*p*-value <0.001) associated with a 5.397 unit increase in a year-over-year increase in the proportion of physically active students. It means that there was nearly a one-to-five unit effect for change in the proportion of physically active students as the change in perception of school connectedness increased from grade to grade (Table 2). As shown in Table 2, the same directionality was observed for all school climate measures except cultural acceptance and peer victimization. However, the effect of the perception of school climate on physical activity did not differ significantly by gender (Appendix E1). Appendix Table E1 presents the full results summarized in Table 2.

**Table 2:** Impact of year-to-year changes in school climate measure on year-to-year changes in the proportion of physically active students.

School climate measures	Year-to-year changes in proportion of physically active students^a^
School connectedness	5.397***
School support environment	5.284***
Peer social support	5.179***
Physical environment	3.563***
School safety	2.951**
Adult social support	2.224*
Cultural acceptance	1.145
Peer victimization	0.573

*p < 0.05, **p < 0.01, ***p < 0.001.

a The outcome variable is the change in proportion of physically active students, the predictor of interest is the changes in school climate perception and includes a two-way interaction of changes in school climate perception with male/female. All models include year fixed effect, and the standard errors are clustered at the school level. All models were run separately for each school climate measure.

## 4 Discussion

We used 5 years (2016 to 2020) of state population-based self-reported data from middle and high school students in a state with established differences in physically active male and female students. The study objective was to identify the impact of changing perceptions of school climate on changing physical activity levels. We found a decrease in the proportion of physically active students as students advanced in grade, with a statistically significantly higher decline among female students compared to male students. Similarly, we found that the improved perception of school climate as students’ progress through the school system, i.e., moving to higher levels, improves physical activity, as evidenced by the significant positive association of grade-to-grade improved perception of school climate measures with grade-to-grade increases in the proportion of physically active students.

### 4.1 Trend in physical activity among middle and high school students

The study findings on the decreasing proportion of physically active students with a higher decline among females, are consistent with previous literature (51, 52). The results also suggest a significant drop in the proportion of physically active students during the transition from middle to high school, which could be due to the fact that in the US, physical activity instruction tends to be more regimented after students leave elementary and middle school (53), and the nature of physical education in higher grades could have accentuated such gender differences (54).

Looking closely, we observe that the boys increase their physical activity in middle school but then drop off as they transition to high school in 9th grade. It could be due to self-sorting by high school students into either a high-intensity physical activity (athletic teams) or only taking the more general physical education to meet the required one credit hour of physical education course requirement in the study state (55). Moreover, the single-credit physical education course can be taken online (56), thus disincentivizing physical activity. It is common among students to take physical education courses online during summer, further plummeting physical activity opportunities for students who are not participating in school sports teams. Girls show a more consistent downward trend without the distortion around school transition points showing an ongoing decline in physical activity during the pre-pubertal and pubertal phases. The finding raises concern for adolescents’ health as they transition to adulthood, and such a decrease in physical activity is associated with a higher risk for cardiovascular disease (CVD), type 2 diabetes, high blood pressure, and obesity (57–60) and is a threat to adolescent mental health (61–63). Interventions to prevent the decline in physical activity at school transition points appear essential to maintaining physical activity, particularly among female adolescents.

### 4.2 Impact of school climate on physical activity

Our finding builds on previous research, including cross-sectional studies and systematic reviews, indicating that the odds of being physically active increased with a positive perception of school climate that includes supportive school environments, school connectedness, peer social support, school physical environments, school safety, and adult social support (21, 64). School connectedness, school support environment, and peer social support were identified as the most important dimensions (effect size > 5), consistent with current literature that shows a strong association between physical activity and school connectedness (65–68), school support environment (64, 69), and peer social support (70–73). Such a phenomenon could be explained by the social development model, which proposes that individual students’ feeling of attachment and commitment (i.e., school connectedness) to the school environment due to support system available at the school promotes standards for positive behavior (such as involvement in exercise or sports) that are consistent with the standards and values of the school around physical activity (74–76). Additionally, as adolescents grow, they spend more time with their friends who share similar preferences toward physical activity, and the Youth Physical Activity Promotion (YPAP) model proposes that such peer social support acts as a reinforcing factor to motivate and ensure continued physical activity participation (77).

Likewise, our study and the current literature suggest that adult social support (71, 73, 78–80) and physical environment (64, 81, 82) are associated with physical activity among adolescents. Availability of school physical environment, such as proper school buildings, access to playgrounds and sports facilities, increases students’ access to resources and equipment. The YPAP model suggests that in a school environment that fosters a motivational environment to build students’ self-efficacy, the availability of such physical resources acts as an enabling factor to engage students in physical activity, further reinforced by the parental support at home and teachers/mentors in school settings (77). Similarly, our study and a 2023 analysis of the National Youth Risk Behavior Survey highlight how safety concerns at school could impact physical activity (83). Any experiences that threaten safety or perceived safety might discourage adolescents from engaging in outdoor physical activities such as walking or playing in parks (84). Such opportunities could be further limited by restrictions from parents due to safety concerns and/or poor physical environments (85, 86).

### 4.3 Implications of findings on public health

The current study and the extent of evidence highlight that investing in improving all components of school climate could help schools reduce the declining trend of physical activity in higher grades. These school climates are interrelated and call for attention to implement holistic interventions that aim to improve the overall school climate. Adolescents spend significant time in school, and the UN sustainable development goals must include schools as an avenue to provide the infrastructure and access to physical activity opportunities to promote their health and well-being.

Besides improved physical activity, improving school climate would have other positive consequences, such as improved academic performance (87–89). Studies have also found the mental health status of adolescents to be likely improved by better school climate, such as school safety, reduced peer victimization and bullying, and school support (90–92). Thus, schools need to pursue evidence-based strategies to improve their climate, such as the CDC’s six strategies to improve school connectedness (93) or published practical guidelines that provide step-by-step procedures to improve climate utilizing the high schools’ existing multitiered framework of outcomes, data, practices, and systems (94). Similarly, schools could focus on improving teacher-student relationships, student–student relationships, and rule clarity as studies have found improved perceptions of students’ relationships with teachers and other peers to reduce peer victimization and bullying and improve school safety (95–97). A longitudinal study among elementary school students found that the change in physical activity depends on school characteristics such as recess time, integrated physical activity programs, free and reduced lunch provision, and geographic location (98).

### 4.4 Strengths and limitations

This study has a few limitations. The survey responses were prone to bias and measurement errors due to the self-reported nature of the questions used to measure physical activity level and school climate perception among middle and high school students. Similarly, we were unable to differentiate between physical activity at home or school; the overall physical activity levels are still indicative of the general physical activity behaviors of the participants. Additionally, the highest level of response on physical activity measure was “4–5 days per week,” which is close to the recommended level but does not align perfectly with the WHO and CDC’s recommended level of physical activity. Thus, caution should be used while interpreting the results on physical activity as the study measures higher vs. lower physical activity levels instead of meeting the physical activity requirement vs. not. Potential confounding due to factors such as the demographic background of the students such as race/ethnicity, socio-economic background, and their parents’ characteristics were not directly controlled for, however, in the US school system the schools serve a neighborhood that has similar race/ethnicity, socio-economic background, and parents’ characteristics and our models cluster standard errors at the school level. The GDoE does not release data with both race/ethnicity and school name to ensure data anonymity, nor does it collect information on digital devices use among adolescents. Similarly, GSHS did not have information on availability and accessibility to resources (especially financially), the rural/urban location of the school, and neighborhood socio-economic status, which could impact the school’s ability to improve its school climate environment. Furthermore, the study was conducted at grade level, as the GSHS was an anonymous survey, and longitudinal follow-up of individual students was not feasible. However, the mandatory participation requirement for public schools allowed for longitudinal follow-up at the grade level for each school, and the homogeneity of the student body served by school districts in the US can safely be assumed (36, 37). Caution needs to be taken while interpreting the findings from this study as the results are drawn from group-level data with the assumption of similar distribution of student body over time, and missing potential confounding variables such as demographic characteristics and school-level contextual factors.

Despite these limitations, the study’s main strength is the longitudinal assessments of the impact of changed perceptions of school climate on changes in the proportion of physically active students from population-based data that requires a 75% participation rate from all middle and high schools of GA (99). Previous studies have either conducted longitudinal assessments with a single school climate measure only or are limited to cross-sectional studies, whereas we incorporate recent data to study all school climate measures. Future longitudinal studies at the individual level that accounts for students’ socio-demographic characteristics (such as social origin) and behavioral characteristics, along with school-level contextual factors (such as geographical location of schools, access to resources, and neighborhood characteristics) are needed to provide robust causal estimate on the impact of school climate on physical activity and assess the presence of any heterogeneous treatment effect by sex, race/ethnicity or school-level characteristics. Additionally, future studies should also strive to measure the physical activity level in detail, such as the level of energy expended, to assess the impact of school climate on different levels of physical activity.

## 5 Conclusion

The study showed a declining trend in the proportion of physically active students as they advanced in grade, particularly among females. Moreover, our study suggests that improvement in school climate will help increase physical activity among students as they progress to higher grades. This connection underscores the importance of fostering positive school climates to mitigate the decline in physical activity levels. Schools should implement evidence-based interventions to improve the school climate and promote physical activity in higher grades for overall adolescent health.

## Data Availability

The study utilized publicly available Georgia Student Health Survey 2.0 data, available to request at https://georgiainsights.gadoe.org/contact-request-data/. The data that supports the findings of this study are available upon reasonable request to the corresponding author.

## References

[1] WHO. Global action plan on physical activity 2018–2030: more active people for a healthier world. Geneva: World Health Organization (2018).

[2] WHO. WHO guidelines on physical activity and sedentary behaviour. Geneva: World Health Organization (2020).

[3] Bielemann RM Martinez-Mesa J Gigante DP. Physical activity during life course and bone mass: a systematic review of methods and findings from cohort studies with young adults. BMC Musculoskelet Disord. (2013) 14:77. doi: 10.1186/1471-2474-14-77, PMID: 23497066 PMC3599107

[4] Chaput JP Willumsen J Bull F Chou R Ekelund U Firth J. 2020 who guidelines on physical activity and sedentary behaviour for children and adolescents aged 5-17 years: summary of the evidence. Int J Behav Nutr Phys Act. (2020) 17:141. doi: 10.1186/s12966-020-01037-z, PMID: 33239009 PMC7691077

[5] He JP Paksarian D Merikangas KR. Physical activity and mental disorder among adolescents in the United States. J Adolesc Health. (2018) 63:628–35. doi: 10.1016/j.jadohealth.2018.05.030, PMID: 30170937

[6] Oliveira RG Guedes DP. Physical activity, sedentary behavior, cardiorespiratory fitness and metabolic syndrome in adolescents: systematic review and meta-analysis of observational evidence. PLoS One. (2016) 11:e0168503. doi: 10.1371/journal.pone.0168503, PMID: 27997601 PMC5173371

[7] Guthold R Stevens GA Riley LM Bull FC. Global trends in insufficient physical activity among adolescents: a pooled analysis of 298 population-based surveys with 1·6 million participants. Lancet Child Adolesc Health. (2020) 4:23–35. doi: 10.1016/s2352-4642(19)30323-2, PMID: 31761562 PMC6919336

[8] Merlo CL Jones SE Michael SL Chen TJ Sliwa SA Lee SH. Dietary and physical activity behaviors among high school students-youth risk behavior survey, United States, 2019. MMWR Suppl. (2020) 69:64–76. doi: 10.15585/mmwr.su6901a8, PMID: 32817612 PMC7440200

[9] Armstrong S Wong CA Perrin E Page S Sibley L Skinner A. Association of physical activity with income, race/ethnicity, and sex among adolescents and young adults in the United States: findings from the National Health and nutrition examination survey, 2007-2016. JAMA Pediatr. (2018) 172:732–40. doi: 10.1001/jamapediatrics.2018.1273, PMID: 29889945 PMC6142913

[10] Telford RM Telford RD Olive LS Cochrane T Davey R. Why are girls less physically active than boys? Findings from the look longitudinal study. PLoS One. (2016) 11:e0150041. doi: 10.1371/journal.pone.0150041, PMID: 26960199 PMC4784873

[11] Llorente-Cantarero FJ Aguilar-Gómez FJ Anguita-Ruiz A Rupérez AI Vázquez-Cobela R Flores-Rojas K. Changes in physical activity patterns from childhood to adolescence: Genobox longitudinal study. Int J Environ Res Public Health. (2020) 17:7227. doi: 10.3390/ijerph17197227, PMID: PMC7579043 33023228

[12] Goldfield GS Henderson K Buchholz A Obeid N Nguyen H Flament MF. Physical activity and psychological adjustment in adolescents. J Phys Act Health. (2011) 8:157–63. doi: 10.1123/jpah.8.2.157, PMID: 21415442

[13] Liu M Zhang J Kamper-DeMarco KE Hu E Yao S. Associations of moderate-to-vigorous physical activity with psychological problems and suicidality in Chinese high school students: a cross-sectional study. PeerJ. (2020) 8:e 8775. doi: 10.7717/peerj.8775, PMID: 32257640 PMC7102502

[14] Fagan MJ Duncan MJ Bedi RP Puterman E Leatherdale ST Faulkner G. Physical activity and substance use among Canadian adolescents: examining the moderating role of school connectedness. Front Public Health. (2022) 10:889987. doi: 10.3389/fpubh.2022.889987, PMID: 36438291 PMC9686278

[15] Fenesi B Graham JD Crichton M Ogrodnik M Skinner J. Physical activity in high school classrooms: a promising avenue for future research. Int J Environ Res Public Health. (2022) 19:688. doi: 10.3390/ijerph19020688, PMID: 35055510 PMC8776126

[16] Schunk DH DiBenedetto MK. Motivation and social cognitive theory. Contemp Educ Psychol. (2020) 60:101832. doi: 10.1016/j.cedpsych.2019.101832

[17] Owen M Kerner C Newson L Noonan R Curry W Kosteli M-C. Investigating adolescent girls' perceptions and experiences of school-based physical activity to inform the girls' peer activity intervention study. J Sch Health. (2019) 89:730–8. doi: 10.1111/josh.12812, PMID: 31257606

[18] Granero-Gallegos A Gómez-López M Rodríguez-Suárez N Abraldes JA Alesi M Bianco A. Importance of the motivational climate in goal, enjoyment, and the causes of success in handball players. Front Psychol. (2017) 8:2081. doi: 10.3389/fpsyg.2017.02081, PMID: 29250011 PMC5717397

[19] Wu X Gai X Yu T Yu H Zhang Y. Perceived motivational climate and stages of exercise behavior change: mediating roles of motivation within and beyond physical education class. Front Psychol. (2021) 12:737461. doi: 10.3389/fpsyg.2021.737461, PMID: 34759869 PMC8573023

[20] van Sluijs EMF Ekelund U Crochemore-Silva I Guthold R Ha A Lubans D. Physical activity Behaviours in adolescence: current evidence and opportunities for intervention. Lancet. (2021) 398:429–42. doi: 10.1016/s0140-6736(21)01259-9, PMID: 34302767 PMC7612669

[21] Rajbhandari-Thapa J Metzger I Ingels J Thapa K Chiang K. School climate-related determinants of physical activity among high school girls and boys. J Adolesc. (2022) 94:642–55. doi: 10.1002/jad.12052, PMID: 35466440

[22] Barbosa A Whiting S Simmonds P Scotini Moreno R Mendes R Breda J. Physical activity and academic achievement: an umbrella review. Int J Environ Res Public Health. (2020) 17:5972. doi: 10.3390/ijerph17165972, PMID: 32824593 PMC7460146

[23] Berkowitz R. School matters: the contribution of positive school climate to equal educational opportunities among ethnocultural minority students. Youth Soc. (2020) 54:372–96. doi: 10.1177/0044118X20970235

[24] Fritz J Cöster ME Rosengren BE Karlsson C Karlsson MK. Daily school physical activity improves academic performance. Sports (Basel). (2020) 8:83. doi: 10.3390/sports8060083, PMID: 32512691 PMC7353619

[25] Gall S Adams L Joubert N Ludyga S Müller I Nqweniso S. Effect of a 20-week physical activity intervention on selective attention and academic performance in children living in disadvantaged neighborhoods: a cluster randomized control trial. PLoS One. (2018) 13:e0206908. doi: 10.1371/journal.pone.0206908, PMID: 30408073 PMC6224098

[26] García-Hermoso A Hormazábal-Aguayo I Fernández-Vergara O González-Calderón N Russell-Guzmán J Vicencio-Rojas F. A before-school physical activity intervention to improve cognitive parameters in children: the active-start study. Scand J Med Sci Sports. (2020) 30:108–16. doi: 10.1111/sms.13537, PMID: 31410887

[27] Graham DJ Lucas-Thompson RG O’Donnell MB. Jump in! An investigation of school physical activity climate, and a pilot study assessing the acceptability and feasibility of a novel tool to increase activity during learning. Front Public Health. (2014) 2:58. doi: 10.3389/fpubh.2014.00058 PMC4035734 24904919

[28] Delgado-Floody P Cristi-Montero C Jerez-Mayorga D Ruiz-Ariza A Guzmán-Guzmán IP Álvarez C. Exploring the mediating role of promoting school physical activity on the relationship between low socioeconomic status and academic achievement and school climate: evidence from 4, 990 Chilean schools. Front Public Health. (2024) 12:1426108. doi: 10.3389/fpubh.2024.1426108, PMID: 38903576 PMC11188408

[29] Ferreira Silva RM Mendonça CR Azevedo VD Raoof Memon A Noll PRES Noll M. Barriers to high school and university students’ physical activity: a systematic review. PLoS One. (2022) 17:e0265913. doi: 10.1371/journal.pone.0265913, PMID: 35377905 PMC8979430

[30] Hu D Zhou S Crowley-McHattan ZJ Liu Z. Factors that influence participation in physical activity in school-aged children and adolescents: a systematic review from the social ecological model perspective. Int J Environ Res Public Health. (2021) 18:3147. doi: 10.3390/ijerph18063147, PMID: 33803733 PMC8003258

[31] CDC. Physical activity for different groups (2021). Available online at: https://www.cdc.gov/physicalactivity/basics/age-chart.html (Accessed on 10/10/2022)

[32] Loux T Matusik M Hamzic A. Trends in U.S. adolescent physical activity and obesity: a 20-year age-period-cohort analysis. Pediatr Obes. (2023) 18:e12996. doi: 10.1111/ijpo.12996, PMID: 36517961

[33] Hodges J Benefield C. Georgia Student Health Survey (Gshs). (2025). Available online at: https://dcconference.gadoe.org/Documents/FY2020/SEA/Georgia%20Student%20Health%20Survey.pdf (Accessed on 2025 May 26)

[34] GDoE. Using the Georgia Student Health Survey to Understand Student Trends: Office of Whole CHild Supports, Georgia Department of Education (2021). Available online at: https://www.gadoe.org/wholechild/GSHS-II/Documents/GSHS%20and%20Wellness%20Survey%20Overview_9.14.2021.pdf?csf=1&e=syN4cj (Accessed on 2023 February 27)

[35] Ingels JB Thapa K Shrestha S Rajbhandari-Thapa J. Cigarette and electronic vapor product use among high school students in Georgia, 2015-2018. Prev Med Rep. (2020) 19:101140. doi: 10.1016/j.pmedr.2020.101140, PMID: 32612907 PMC7322347

[36] Owens A Rich P. Little boxes all the same? Racial-ethnic segregation and educational inequality across the urban-suburban divide. RSF Russell Sage Found J Soc Sci. (2023) 9:26–54. doi: 10.7758/rsf.2023.9.2.02

[37] United States Government Accountability Office. K12 education student population has significantly diversified, but many schools remain divided along racial, ethnic, and economic lines. US: United States Government Accountability Office (2022).

[38] Georgia Secretary of State. Release 82 of the Official Code of Georgia, Title 20 Education: 20-2-291. (2021). Available online at: https://unicourt.github.io/cic-code-ga/transforms/ga/ocga/r82/gov.ga.ocga.title.20.html#t20c02a06p13s20-2-291 (Accessed December 12, 2023).

[39] Education Week. Map: coronavirus and school closures (2020, march 6) (2020). Available online at: https://www.edweek.org/leadership/map-coronavirus-and-school-closures-in-2019-2020/2020/03 (Accessed on 2023 December 17)

[40] Greer J Thapa K McNulty J Thapa JR. Parental school involvement on physical activity and screen time among middle and high school students. J Ga Public Health Assoc. (2021) 8:3. doi: 10.20429/jgpha.2021.080303

[41] Seidu A-A Ahinkorah BO Agbaglo E Darteh EKM Ameyaw EK Budu E. Are senior high school students in Ghana meeting who’s recommended level of physical activity? Evidence from the 2012 global school-based student health survey data. PLoS One. (2020) 15:e0229012. doi: 10.1371/journal.pone.0229012, PMID: 32050008 PMC7015424

[42] Collins TA LST P Jesslynn RN FJ A Scott MN. No safe space: school climate experiences of black boys with and without emotional and behavioral disorders. Sch Psychol Rev. (2023) 52:250–63. doi: 10.1080/2372966X.2021.2021783

[43] Kittelman A La Salle TP Mercer SH McIntosh K. Identifying profiles of school climate in high schools. Sch Psychol Forum. (2024) 39:50–60. doi: 10.1037/spq0000553, PMID: 37141041

[44] La Salle TP Wang C Parris L Brown JA. Associations between school climate, suicidal thoughts, and behaviors and ethnicity among middle school students. Psychol Sch. (2017) 54:1294–301. doi: 10.1002/pits.22078

[45] Parris L Neves JR La Salle T. School climate perceptions of ethnically diverse students: does school diversity matter? Sch Psychol Int. (2018) 39:625–45. doi: 10.1177/0143034318798419

[46] Salle TL George HP McCoach DB Polk T Evanovich LL. An examination of school climate, victimization, and mental health problems among middle school students self-identifying with emotional and behavioral disorders. Behav Disord. (2018) 43:383–92. doi: 10.1177/0198742918768045

[47] Wang C Salle T Wu C Do K Sullivan K. School climate and parental involvement buffer the risk of peer victimization on suicidal thoughts and behaviors among Asian American middle school students. Asian Am J Psychol. (2018) 9:296–307. doi: 10.1037/aap0000138

[48] La Salle TP McCoach DB Meyers J. Examining measurement invariance and perceptions of school climate across gender and race and ethnicity. J Psychoeduc Assess. (2021) 39:800–15. doi: 10.1177/07342829211023717

[49] La Salle TP Meyers J. The Georgia Student Health Survey 2.0. Atlanta, GA: Georgia Department of Education (2014).

[50] Allison PD. Change scores as dependent variables in regression analysis. Sociol Methodol. (1990) 20:93–114. doi: 10.2307/271083

[51] Kann L McManus T Harris WA Shanklin SL Flint KH Queen B. Youth risk behavior surveillance-United States, 2017. MMWR Surveill Summ. (2018) 67:1–114. doi: 10.15585/mmwr.ss6708a1, PMID: 29902162 PMC6002027

[52] Lee BY Adam A Zenkov E Hertenstein D Ferguson MC Wang PI. Modeling the economic and health impact of increasing children’s physical activity in the United States. Health Aff. (2017) 36:902–8. doi: 10.1377/hlthaff.2016.1315, PMID: 28461358 PMC5563819

[53] Corbin CB. Conceptual physical education: a course for the future. J Sport Health Sci. (2021) 10:308–22. doi: 10.1016/j.jshs.2020.10.004, PMID: 33068747 PMC7554458

[54] Guerrero MA Guerrero Puerta L. Advancing gender equality in schools through inclusive physical education and teaching training: a systematic review. Sociol Sci. (2023) 13:64. doi: 10.3390/soc13030064

[55] GDoE. Rule 160–4-2-.48: high school graduation requirements for students enrolling in the ninth grade for the first time in the 2008–09 school year and subsequent years (2020). Available online at: https://rules.sos.ga.gov/gac/160-4-2-.48 (Accessed on 2024 January 12)

[56] GDoE. (2010). Georgia high school graduation requirements: preparing students for success. Available online at: https://www.gadoe.org/Curriculum-Instruction-and-Assessment/Curriculum-and-Instruction/Documents/Grad%20Guidance%20Doc%20REVISED%20-%20January%2006,%202010.pdf (Accessed on 2024 January 12)

[57] Ashcraft KA Peace RM Betof AS Dewhirst MW Jones LW. Efficacy and mechanisms of aerobic exercise on Cancer initiation, progression, and metastasis: a critical systematic review of in vivo preclinical data. Cancer Res. (2016) 76:4032–50. doi: 10.1158/0008-5472.Can-16-0887, PMID: 27381680 PMC5378389

[58] Herting MM Chu X. Exercise, cognition, and the adolescent brain. Birth Defects Res. (2017) 109:1672–9. doi: 10.1002/bdr2.1178, PMID: 29251839 PMC5973814

[59] Janssen I Leblanc AG. Systematic review of the health benefits of physical activity and fitness in school-aged children and youth. Int J Behav Nutr Phys Act. (2010) 7:40. doi: 10.1186/1479-5868-7-40, PMID: 20459784 PMC2885312

[60] Stanford KI Goodyear LJ. Exercise and type 2 diabetes: molecular mechanisms regulating glucose uptake in skeletal muscle. Adv Physiol Educ. (2014) 38:308–14. doi: 10.1152/advan.00080.2014, PMID: 25434013 PMC4315445

[61] Andermo S Hallgren M Nguyen T-T-D Jonsson S Petersen S Friberg M. School-related physical activity interventions and mental health among children: a systematic review and meta-analysis. Sports Med-Open. (2020) 6:25. doi: 10.1186/s40798-020-00254-x, PMID: 32548792 PMC7297899

[62] Chi X Liang K Chen ST Huang Q Huang L Yu Q. Mental health problems among Chinese adolescents during the Covid-19: the importance of nutrition and physical activity. Int J Clin Health Psychol. (2021) 21:100218. doi: 10.1016/j.ijchp.2020.100218, PMID: 33391373 PMC7759093

[63] Marconcin P Werneck AO Peralta M Ihle A Gouveia ÉR Ferrari G. The association between physical activity and mental health during the first year of the Covid-19 pandemic: a systematic review. BMC Public Health. (2022) 22:209. doi: 10.1186/s12889-022-12590-6, PMID: 35101022 PMC8803575

[64] Morton KL Atkin AJ Corder K Suhrcke M van Sluijs EMF. The school environment and adolescent physical activity and sedentary behaviour: a mixed-studies systematic review. Obes Rev. (2016) 17:142–58. doi: 10.1111/obr.12352, PMID: 26680609 PMC4914929

[65] Faulkner GEJ Adlaf EM Irving HM Allison KR Dwyer J. School disconnectedness: identifying adolescents at risk in Ontario, Canada. J Sch Health. (2009) 79:312–8. doi: 10.1111/j.1746-1561.2009.00415.x, PMID: 19527413

[66] Michael SL Li J Sliwa S Cornett K Hertz M. Association between adolescent self-reported physical activity behaviors and feeling close to people at school during the Covid-19 pandemic. Am J Lifestyle Med. (2024) 18:364–75. doi: 10.1177/15598276231157324, PMID: 38737878 PMC9941458

[67] Trinh L Wong B Faulkner GE. The independent and interactive associations of screen time and physical activity on mental health, school connectedness and academic achievement among a population-based sample of youth. J Can Acad Child Adolesc Psychiatry. (2015) 24:17–24. doi: 10.1111/j.1467-789X.2005.00176.x, PMID: 26336376 PMC4357330

[68] Weatherson KA O'Neill M Lau EY Qian W Leatherdale ST Faulkner GEJ. The protective effects of school connectedness on substance use and physical activity. J Adolesc Health. (2018) 63:724–31. doi: 10.1016/j.jadohealth.2018.07.002, PMID: 30269908

[69] Chen R Wang L Wang B Zhou Y. Motivational climate, need satisfaction, self-determined motivation, and physical activity of students in secondary school physical education in China. BMC Public Health. (2020) 20:1687. doi: 10.1186/s12889-020-09750-x, PMID: 33172411 PMC7657358

[70] Chen H Sun H Dai J. Peer support and adolescents’ physical activity: the mediating roles of self-efficacy and enjoyment. J Pediatr Psychol. (2017) 42:569–77. doi: 10.1093/jpepsy/jsw103, PMID: 28158660

[71] Haidar A Ranjit N Archer N Hoelscher DM. Parental and peer social support is associated with healthier physical activity behaviors in adolescents: a cross-sectional analysis of Texas school physical activity and nutrition (Tx span) data. BMC Public Health. (2019) 19:640. doi: 10.1186/s12889-019-7001-0, PMID: 31132999 PMC6537420

[72] Mendonça G Farias Júnior JC. Physical activity and social support in adolescents: analysis of different types and sources of social support. J Sports Sci. (2015) 33:1942–51. doi: 10.1080/02640414.2015.1020842 25751023

[73] Mendonça G Cheng LA Mélo EN de Farias Júnior JC. Physical activity and social support in adolescents: a systematic review. Health Educ Res. (2014) 29:822–39. doi: 10.1093/her/cyu017, PMID: 24812148

[74] Catalano RF Oesterle S Fleming CB Hawkins JD. The importance of bonding to school for healthy development: findings from the social development research group. J Sch Health. (2004) 74:252–61. doi: 10.1111/j.1746-1561.2004.tb08281.x 15493702

[75] Chapman RL Buckley L Sheehan M Shochet I. School-based programs for increasing connectedness and reducing risk behavior: a systematic review. Educ Psychol Rev. (2013) 25:95–114. doi: 10.1007/s10648-013-9216-4

[76] Hawkins JD Catalano RF Kosterman R Abbott R Hill KG. Preventing adolescent health-risk behaviors by strengthening protection during childhood. Arch Pediatr Adolesc Med. (1999) 153:226–34. doi: 10.1001/archpedi.153.3.226, PMID: 10086398

[77] Welk GJ. The youth physical activity promotion model: a conceptual bridge between theory and practice. Quest. (1999) 51:5–23. doi: 10.1080/00336297.1999.10484297

[78] Eather N Morgan PJ Lubans DR. Social support from teachers mediates physical activity behavior change in children participating in the Fit-4-fun intervention. Int J Behav Nutr Phys Act. (2013) 10:68. doi: 10.1186/1479-5868-10-68, PMID: 23714651 PMC3672071

[79] Pluta B Korcz A Krzysztoszek J Bronikowski M Bronikowska M. Associations between adolescents’ physical activity behavior and their perceptions of parental, peer and teacher support. Arch Public Health. (2020) 78:106. doi: 10.1186/s13690-020-00490-3, PMID: 33110599 PMC7585189

[80] Shen B Li W Sun H Rukavina PB. The influence of inadequate teacher-to-student social support on amotivation of physical education students. J Teach Phys Educ. (2010) 29:417–32. doi: 10.1123/jtpe.29.4.417

[81] Hawkins GT Chung CS Hertz MF Antolin N. The school environment and physical and social-emotional well-being: implications for students and school employees. J Sch Health. (2023) 93:799–812. doi: 10.1111/josh.13375, PMID: 37670600

[82] Crooks N Alston L Nichols M Bolton KA Allender S Fraser P. Association between the school physical activity environment, measured and self-reported student physical activity and active transport Behaviours in Victoria, Australia. Int J Behav Nutr Phys Act. (2021) 18:79. doi: 10.1186/s12966-021-01151-6, PMID: 34158052 PMC8220765

[83] Cornett K Michael S Sliwa S Chen T Kissler C McKinnon I. Physical activity behaviors and negative safety and violence experiences among high school students-youth risk behavior survey, United States, 2023. MMWR Suppl. (2024) 73:94–103. doi: 10.15585/mmwr.su7304a11, PMID: 39378265 PMC11559679

[84] Constable Fernandez C Patalay P Vaughan L Church D Hamer M Maddock J. Subjective and objective indicators of Neighbourhood safety and physical activity among Uk adolescents. Health Place. (2023) 83:103050. doi: 10.1016/j.healthplace.2023.103050, PMID: 37348294

[85] Ries AV Gittelsohn J Voorhees CC Roche KM Clifton KJ Astone NM. The environment and urban adolescents' use of recreational facilities for physical activity: a qualitative study. Am J Health Promot. (2008) 23:43–50. doi: 10.4278/ajhp.07043042, PMID: 18785374

[86] Zhu M Teng R Wang C Wang Y He J Yu F. Key environmental factors affecting perceptions of security of night-time walking in neighbourhood streets: a discussion based on fear heat maps. J Transp Health. (2023) 32:101636. doi: 10.1016/j.jth.2023.101636

[87] Berkowitz R Moore H Astor RA Benbenishty R. A research synthesis of the associations between socioeconomic background, inequality, school climate, and academic achievement. Rev Educ Res. (2016) 87:425–69. doi: 10.3102/d0034654316669821

[88] Cuellar MJ Coyle S Weinreb KS. Dealing with the day-to-day: harnessing school climate to address the effects of student victimization on academic performance. Psychol Sch. (2021) 58:1799–815. doi: 10.1002/pits.22560

[89] Wang W Vaillancourt T Brittain HL McDougall P Krygsman A Smith D. School climate, peer victimization, and academic achievement: results from a multi-informant study. Sch Psychol Q. (2014) 29:360–77. doi: 10.1037/spq0000084, PMID: 25198617

[90] Franco K Baumler E Torres ED Lu Y Wood L Temple JR. The link between school climate and mental health among an ethnically diverse sample of middle school youth. Curr Psychol. (2023) 42:18488–98. doi: 10.1007/s12144-022-03016-y, PMID: 35370383 PMC8965219

[91] Leurent B Dodd M Allen E Viner R Scott S Bonell C. Is positive school climate associated with better adolescent mental health? Longitudinal study of young people in England. SSM Ment Health. (2021) 1:33. doi: 10.1016/j.ssmmh.2021.100033, PMID: 34957422 PMC8654679

[92] Lombardi E Traficante D Bettoni R Offredi I Giorgetti M Vernice M. The impact of school climate on well-being experience and school engagement: a study with high-school students. Front Psychol. (2019) 10:10. doi: 10.3389/fpsyg.2019.02482, PMID: 31749747 PMC6848455

[93] CDC. School connectedness; strategies for increasing protective factors among youth. Centers for Disease C, Prevention, National Center for Chronic Disease P, Health Promotion DoA, School H. US: Center for Disease Control and Prevention (2009).

[94] Van Lone J Freeman J LaSalle T Gordon L Polk T Rocha Neves J. A practical guide to improving school climate in high schools. Interv Sch Clin. (2019) 55:39–45. doi: 10.1177/1053451219832988

[95] Aldridge JM McChesney K Afari E. Associations between school climate and student life satisfaction: resilience and bullying as mediating factors. Learn Environ Res. (2020) 23:129–50. doi: 10.1007/s10984-019-09296-9

[96] Ferrer- Cascales R Albaladejo- Blázquez N Sánchez- San Segundo M Portilla- Tamarit I Lordan O Ruiz- Robledillo N. Effectiveness of the TEI program for bullying and cyberbullying reduction and school climate improvement. Int J Environ Res Public Health. (2019) 16:580. doi: 10.3390/ijerph16040580 PMC6406958 30781543

[97] Lewno-Dumdie BM Mason BA Hajovsky DB Villeneuve EF. Student-report measures of school climate: a dimensional review. Sch Ment Heal. (2020) 12:1–21. doi: 10.1007/s12310-019-09340-2

[98] Rajbhandari-Thapa J Ingels J Thapa K Davis M Corso P. Longitudinal evaluation of the impact of school characteristics on changes in physical activity opportunities. PLoS One. (2020) 15:e0228716. doi: 10.1371/journal.pone.0228716, PMID: 32027725 PMC7004365

[99] GDoE. (2019). School climate star ratings data calculation guide for principals and district users (2019). Available online at: https://www.gadoe.org/wholechild/Documents/2019%20School%20Climate%20Star%20Rating%20Calculation%20Guide_October%202019.pdf (Accessed on 2024 January 12)

