# Supplementary material for: Physical activity level among male and female middle to high school students and impact of perceived school climate: a longitudinal analysis
Source: Front Public Health. 2025 Jul 18;13:1582693. doi: 10.3389/fpubh.2025.1582693 (PMC12313673; doi:10.3389/fpubh.2025.1582693)
Supplement: Supplementary file 1 [file Table_1.docx]

Supplementary Material

# Appendix A

**Table A1: Measures of school climate and corresponding questions as asked in the Georgia Student Health Survey (2016 to 2020)**

| **Measures** | **Questions (answer options in parentheses)** | **Changes made in 2019 onwards** | **Questions Included in the Study** |
| --- | --- | --- | --- |
| **School connectedness** | Q1: I like school, Q2: Most days I look forward to going to school, Q3: I feel like I fit in at my school, Q4: I feel successful at school, Q5: I feel connected to others at school (strongly disagree/somewhat disagree/somewhat agree/ strongly agree) | None | Q1 to Q5 |
| **Peer social support** | Q6: I get along with other students and school, Q7: I know a student at my school that I can talk to if I need help (e.g., with homework, class assignments, and projects), Q8: I know a student at my school that I can talk to if I am feeling sad or down, Q9: I have a group of friends at school that I have fun with and are nice to me, Q10: Students in my school are welcoming to new students. (strongly disagree/somewhat disagree/somewhat agree/strongly agree) | Q8 and Q9 were removed from 2019 onwards | Q6, Q7, Q10 |
| **Adult social support** | Q11: Teachers treat me with respect, Q12: Adults in this school treat all students with respect, Q13: All students are treated fairly by the adults in my school, Q14: Teachers treat all students fairly (strongly disagree/somewhat disagree/somewhat agree/strongly agree) | None | Q11 to Q14 |
| **Cultural acceptance** | Q15: Students at my school treat each other with respect, Q16: Students treat one another fairly, Q17: Students show respect to other students regardless of their academic ability, Q18: Students at this school are treated fairly by other students regardless of race, ethnicity, or culture, Q19: All students in my school are treated fairly regardless of their appearance (strongly disagree/somewhat disagree/somewhat agree/strongly agree) | None | Q15 to Q19 |
| **Physical environment** | Q28: My school building is well-maintained,  Q29: My textbooks are up to date and in good condition.  Q30: Teachers in my school keep their classrooms clean and organized.  Q31: Students in my school take pride in keeping our school building (e.g., bathrooms, classrooms, lockers) in good condition (selected based on connection with Physical activity) (strongly disagree/somewhat disagree/somewhat agree/strongly agree) | None | Q28 to Q31 |
| **School Safety** | Q32: I have felt unsafe at school or on my way to or from school*, Q33: I have worried about other students hurting me*, Q34: I feel safe in my school, Q35: I have been concerned about my physical safety at school*, Q36: Students at my school fight a lot*, Q37: I have been involved in a fight at school*, Q38: I have observed a fight at school* (strongly disagree, somewhat disagree, somewhat agree, strongly agree) | Q37 and Q38 were removed from 2019 onwards  2019 onwards Q34 shifted to peer victimization | Q32 to Q36 |
| **Peer victimization** | Q39: I have been bullied or threatened by other students*, Q40: I have been picked on or teased at school*, Q41: have received a threatening or harassing e‐mail from other students*, Q42: I have received threatening or harassing text messages from other students (SMS) *, Q43: I have been mocked, tormented, or harassed on a social networking site (e.g., Facebook, Twitter) by other students*, Q44: Someone has bullied or picked on me by pushing, hitting, or kicking me*, Q45: Someone has bullied or picked on me by making fun of me, yelling at me, or saying something mean to me* (Never/Once or twice/a few times/many times/every day) | Q41 was removed from 2019 onwards | Q39, Q40, Q42 to Q45 |
| **School support environment** | Q87: I feel my school has high standards for achievement, Q88: My school sets clear rules for behavior, Q89: The behaviors in my classroom allow the teacher to teach so I can learn, Q90: Students are frequently recognized for good behavior, Q91: I know an adult at school that I can talk with if I need help, Q92: I know what to do if there is an emergency at my school, Q93: I would help someone who was being bullied (Strongly Disagree/Somewhat Disagree/Somewhat Agree/Strongly Agree) | Q93 was removed from 2019 onwards | Q87 to Q92 |

Note: * Reordered Responses

# Appendix B

**Table B1: Summary of School Climate Factors from Factor Analysis**

| **School Year** | **Measures** | **No. of Items** | **Cronbach's alpha** | **Min.** | **Max.** | **No. of Eigenvalue > 1** | **Eigenvalue** | **Proportion of Variance (%)** |
| --- | --- | --- | --- | --- | --- | --- | --- | --- |
| **2016** | **School connectedness** | 5 | 0.8 | -2.76 | 1.43 | 1 | 2.28 | 44.5 |
|  | **Peer social support** | 3 | 0.67 | -2.85 | 1.04 | 1 | 1.22 | 40.7 |
|  | **Adult social support** | 4 | 0.92 | -2.13 | 1.27 | 1 | 2.96 | 74 |
|  | **Cultural acceptance** | 5 | 0.91 | -2.01 | 1.69 | 1 | 3.32 | 66.3 |
|  | **Physical environment** | 4 | 0.8 | -2.16 | 1.44 | 1 | 2.04 | 51 |
|  | **School Safety** | 5 | 0.71 | -2.39 | 1.11 | 1 | 1.78 | 35.6 |
|  | **Peer victimization** | 6 | 0.88 | -5.81 | 0.44 | 1 | 3.47 | 57.8 |
|  | **School support environment** | 6 | 0.85 | -2.86 | 1.13 | 1 | 3.02 | 50.3 |
| **2017** | **School connectedness** | 5 | 0.8 | -2.78 | 1.44 | 1 | 2.24 | 44.8 |
|  | **Peer social support** | 3 | 0.67 | -2.89 | 1.04 | 1 | 1.224 | 40.8 |
|  | **Adult social support** | 4 | 0.92 | -2.17 | 1.24 | 1 | 2.97 | 74.3 |
|  | **Cultural acceptance** | 5 | 0.91 | -2.04 | 1.67 | 1 | 3.32 | 66.5 |
|  | **Physical environment** | 4 | 0.81 | -2.19 | 1.42 | 1 | 2.05 | 51.2 |
|  | **School Safety** | 5 | 0.72 | -2.44 | 1.08 | 1 | 1.81 | 36.3 |
|  | **Peer victimization** | 6 | 0.88 | -5.99 | 0.43 | 1 | 3.47 | 57.7 |
|  | **School support environment** | 6 | 0.85 | -2.91 | 1.12 | 1 | 3.01 | 50.1 |
| **2018** | **School connectedness** | 5 | 0.79 | -2.69 | 1.45 | 1 | 2.12 | 42.4 |
|  | **Peer social support** | 3 | 0.64 | -2.83 | 1 | 1 | 1.15 | 38.3 |
|  | **Adult social support** | 4 | 0.91 | -2.14 | 1.22 | 1 | 2.89 | 72.3 |
|  | **Cultural acceptance** | 5 | 0.89 | -2.00 | 1.64 | 1 | 3.19 | 63.8 |
|  | **Physical environment** | 4 | 0.79 | -2.39 | 1.28 | 1 | 1.96 | 49 |
|  | **School Safety** | 5 | 0.73 | -2.37 | 1.08 | 1 | 1.86 | 37.1 |
|  | **Peer victimization** | 6 | 0.89 | -5.51 | 0.44 | 1 | 3.65 | 60.9 |
|  | **School support environment** | 6 | 0.83 | -2.93 | 1.13 | 1 | 2.69 | 45 |
| **2019** | **School connectedness** | 5 | 0.79 | -2.64 | 1.49 | 1 | 2.13 | 42.6 |
|  | **Peer social support** | 3 | 0.65 | -2.81 | 1.01 | 1 | 1.16 | 38.6 |
|  | **Adult social support** | 4 | 0.91 | -2.23 | 1.2 | 1 | 2.85 | 71.3 |
|  | **Cultural acceptance** | 5 | 0.88 | -2.06 | 1.63 | 1 | 3.01 | 60.1 |
|  | **Physical environment** | 4 | 0.79 | -2.45 | 1.27 | 1 | 1.95 | 48.6 |
|  | **School Safety** | 5 | 0.71 | -2.52 | 0.94 | 1 | 2.01 | 40.1 |
|  | **Peer victimization** | 6 | 0.88 | -5.58 | 0.44 | 1 | 3.45 | 57.5 |
|  | **School support environment** | 6 | 0.82 | -2.87 | 1.19 | 1 | 2.61 | 43.6 |
| **2020** | **School connectedness** | 5 | 0.79 | -2.57 | 1.54 | 1 | 2.18 | 43.5 |
|  | **Peer social support** | 3 | 0.66 | -2.78 | 1.05 | 1 | 1.201 | 40 |
|  | **Adult social support** | 4 | 0.91 | -2.20 | 1.24 | 1 | 2.86 | 71.4 |
|  | **Cultural acceptance** | 5 | 0.89 | -2.05 | 1.7 | 1 | 3.04 | 60.9 |
|  | **Physical environment** | 4 | 0.79 | -2.44 | 1.29 | 1 | 1.95 | 48.8 |
|  | **School Safety** | 5 | 0.71 | -2.51 | 0.95 | 1 | 2.05 | 41 |
|  | **Peer victimization** | 6 | 0.88 | -5.79 | 0.43 | 1 | 3.43 | 57.1 |
|  | **School support environment** | 6 | 0.82 | -2.83 | 1.22 | 1 | 2.62 | 43.6 |

# Appendix C

**Table C1: Physically Active Students (%) by Grade and Survey Year for Female and Male Students**

|  | **Year** | **Grades** | | | | | | | |
| --- | --- | --- | --- | --- | --- | --- | --- | --- | --- |
|  |  | **6** | **7** | **8** | **9** | **10** | **11** | **12** | **Decrease from grade 6^th^ to 12^th^** |
| **Female** | 2016 | 51.58 | 48.16 | 42.63 | 41.10 | 34.76 | 32.50 | 30.88 | 20.70 |
|  | 2017 | 52.03 | 48.00 | 42.89 | 40.88 | 34.61 | 32.97 | 31.37 | 20.66 |
|  | 2018 | 49.36 | 46.29 | 41.68 | 39.68 | 34.02 | 31.40 | 30.53 | 18.84 |
|  | 2019 | 38.20 | 36.46 | 33.59 | 33.73 | 28.44 | 26.08 | 24.18 | 14.02 |
|  | 2020 | 37.09 | 34.68 | 32.38 | 32.46 | 28.35 | 25.79 | 24.12 | 12.97 |
|  | Mean | 45.38 | 42.51 | 38.51 | 37.52 | 32.01 | 29.69 | 28.15 | 17.23 |
| **Male** | 2016 | 62.13 | 63.35 | 63.03 | 61.37 | 58.66 | 56.11 | 54.67 | 7.46 |
|  | 2017 | 62.28 | 63.92 | 63.61 | 60.82 | 57.08 | 56.79 | 53.78 | 8.50 |
|  | 2018 | 59.40 | 60.66 | 60.51 | 58.71 | 55.08 | 53.04 | 50.45 | 8.95 |
|  | 2019 | 46.78 | 49.00 | 49.08 | 47.35 | 45.70 | 42.90 | 40.36 | 6.42 |
|  | 2020 | 47.87 | 48.75 | 48.63 | 47.33 | 44.89 | 43.31 | 40.24 | 7.63 |
|  | Mean | 55.42 | 56.91 | 56.75 | 55.00 | 52.16 | 50.23 | 47.55 | 7.87 |

# Appendix D

**Table D1: Association of Proportion of Physically Active Students with Grade, Male/Female status and Interaction by Year**

| **Components** | **Proportion of Physically Active Students** | | | | |
| --- | --- | --- | --- | --- | --- |
|  | **2016** | **2017** | **2018** | **2019** | **2020** |
| **Female (ref. Male)** | -10.069** | -9.193*** | -8.780*** | -8.285*** | -9.261*** |
| **Ref. (Grade 6)** | 62.433*** | 62.455*** | 59.061*** | 46.943*** | 47.070*** |
| **Grade 7** | 0.702 | 1.125 | 2.501** | 1.299 | 0.653 |
| **Grade 7 * Female** | -4.739*** | -5.652*** | -5.575** | -3.263* | -3.131* |
| **Grade 8** | 0.911 | 1.326 | 1.712 | 1.604 | 1.254 |
| **Grade 8 * Female** | -10.471*** | -11.009*** | -9.745** | -5.545** | -7.046** |
| **Grade 9** | -0.534 | -2.196* | -1.088 | -0.546 | -0.951 |
| **Grade 9 * Female** | -10.780*** | -10.662*** | -9.819** | -5.535** | -6.006** |
| **Grade 10** | -4.294*** | -5.346*** | -3.302*** | -2.306* | -2.499* |
| **Grade 10 * Female** | -13.931*** | -13.170*** | -13.680** | -8.809** | -7.709** |
| **Grade 11** | -5.804*** | -5.549*** | -5.127*** | -5.314*** | -4.829*** |
| **Grade 11 * Female** | -15.058*** | -14.009*** | -13.367** | -6.881** | -7.383** |
| **Grade 12** | -6.730*** | -9.237*** | -8.711*** | -5.217*** | -6.811*** |
| **Grade 12 * Female** | -14.694*** | -12.660*** | -10.334** | -9.079** | -6.464** |

**Note:** *p<0.05; **p<0.01; ; ***p<0.001. Models were run separately for each year. The dependent variable is the proportion of physically active students.

# Appendix E

**Table E1: Impact of changes in school climate measure on changes in the proportion of physically active students**

| **School Climate Measures** | **Year-to-Year Changes in Proportion of Physically Active Students^a^** |
| --- | --- |
| **School Connectedness** |  |
| Intercept | 39.294*** |
| Proportion of physically active students in previous year | -0.613*** |
| Change in perception of school connectedness | 5.397*** |
| Female (reference Male) | -11.288*** |
| Grade (ref. Grade 6-to-Grade 7) |  |
| Grade 7-to-Grade 8 | -1.659*** |
| Grade 8-to-Grade 9 | -6.062*** |
| Grade 9-to-Grade 10 | -6.099*** |
| Grade 10-to-Grade 11 | -6.465*** |
| Grade 11-to-Grade 12 | -7.645*** |
| Change in perception of school connectedness × Female | -1.659 |
| **Peer Social Support** |  |
| Intercept | 39.009*** |
| Proportion of physically active students in previous year | -0.614*** |
| Change in perception of peer social support | 5.179*** |
| Female (reference Male) | -11.266*** |
| Grade (ref. Grade 6-to-Grade 7) |  |
| Grade 7-to-Grade 8 | -1.568*** |
| Grade 8-to-Grade 9 | -5.786** |
| Grade 9-to-Grade 10 | -5.974*** |
| Grade 10-to-Grade 11 | -6.235*** |
| Grade 11-to-Grade 12 | -7.189*** |
| Change in perception of peer social support × Female | -0.501 |
| **Adult Social Support** |  |
| Intercept | 39.371*** |
| Proportion of physically active students in previous year | -0.621*** |
| Change in perception of adult social support | 2.224* |
| Female (reference Male) | -11.459*** |
| Grade (ref. Grade 6-to-Grade 7) |  |
| Grade 7-to-Grade 8 | -1.633*** |
| Grade 8-to-Grade 9 | -5.954*** |
| Grade 9-to-Grade 10 | -6.085*** |
| Grade 10-to-Grade 11 | -6.369*** |
| Grade 11-to-Grade 12 | -7.416*** |
| Change in perception of adult social support × Female | -0.873 |
| **Cultural Acceptance** |  |
| Intercept | 39.371*** |
| Proportion of physically active students in previous year | -0.622*** |
| Change in perception of cultural acceptance | 1.145 |
| Female (reference Male) | -11.451*** |
| Grade (ref. Grade 6-to-Grade 7) |  |
| Grade 7-to-Grade 8 | -1.499*** |
| Grade 8-to-Grade 9 | -5.746** |
| Grade 9-to-Grade 10 | -5.939*** |
| Grade 10-to-Grade 11 | -6.111*** |
| Grade 11-to-Grade 12 | -7.117*** |
| Change in perception of cultural acceptance × Female | -0.613 |
| **Physical Environment** |  |
| Intercept | 39.784*** |
| Proportion of physically active students in previous year | -0.619*** |
| Change in perception of physical environment | 3.563*** |
| Female (reference Male) | -11.411*** |
| Grade (ref. Grade 6-to-Grade 7) |  |
| Grade 7-to-Grade 8 | -1.788*** |
| Grade 8-to-Grade 9 | -6.107*** |
| Grade 9-to-Grade 10 | -6.260*** |
| Grade 10-to-Grade 11 | -6.729*** |
| Grade 11-to-Grade 12 | -7.814*** |
| Change in perception of physical environment × Female | -0.433 |
| **School Safety** |  |
| Intercept | 38.781*** |
| Proportion of physically active students in previous year | -0.619*** |
| Change in perception of school safety | 2.951** |
| Female (reference Male) | -11.352*** |
| Grade (ref. Grade 6-to-Grade 7) |  |
| Grade 7-to-Grade 8 | -1.354*** |
| Grade 8-to-Grade 9 | -5.324** |
| Grade 9-to-Grade 10 | -5.579*** |
| Grade 10-to-Grade 11 | -5.759*** |
| Grade 11-to-Grade 12 | -6.679*** |
| Change in perception of school safety × Female | 0.115 |
| **Peer Victimization** |  |
| Intercept | 39.246*** |
| Proportion of physically active students in previous year | -0.622*** |
| Change in perception of peer victimization | 0.573 |
| Female (reference Male) | -11.354*** |
| Grade (ref. Grade 6-to-Grade 7) |  |
| Grade 7-to-Grade 8 | -1.424*** |
| Grade 8-to-Grade 9 | -5.624*** |
| Grade 9-to-Grade 10 | -5.805*** |
| Grade 10-to-Grade 11 | -5.943*** |
| Grade 11-to-Grade 12 | -6.944*** |
| Change in perception of peer victimization × Female | -2.188 |
| **School Support Environment** |  |
| Intercept | 39.595*** |
| Proportion of physically active students in previous year | -0.612*** |
| Change in perception of school support environment | 5.284*** |
| Female (reference Male) | -11.418*** |
| Grade (ref. Grade 6-to-Grade 7) |  |
| Grade 7-to-Grade 8 | -1.829*** |
| Grade 8-to-Grade 9 | -6.094*** |
| Grade 9-to-Grade 10 | -6.397*** |
| Grade 10-to-Grade 11 | -6.869*** |
| Grade 11-to-Grade 12 | -7.949*** |
| Change in perception of school support environment × Female | -0.846 |

Note: *p<0.05, **p<0.01, ***p<0.001. **^a^**The outcome variable is the change in physical activity, the predictor of interest is the changes in school climate perception, and also includes a two-way interaction of the changes in school climate perception with male/female. All models include year fixed effect, and the standard errors are clustered at the school level. All models were run separately for each school climate measure.
